# The First Observation of Memory Effects in the InfraRed (FT-IR) Measurements: Do Successive Measurements Remember Each Other?

**DOI:** 10.1371/journal.pone.0094305

**Published:** 2014-04-10

**Authors:** Raoul R. Nigmatullin, Sergey I. Osokin, Dumitru Baleanu, Sawsan Al-Amri, Ameer Azam, Adnan Memic

**Affiliations:** 1 Theoretical Physics Department, Kazan Federal University, Kazan, Russia; 2 Chemical and Materials Engineering Department, King Abdulaziz University, Jeddah, Saudi Arabia; 3 Department of Mathematics and Computer Sciences, Cankaya University, Ankara, Turkey; 4 Institute of Space Sciences, Magurele-Bucharest, Romania; 5 Center of Nanotechnology, King Abdulaziz University, Jeddah, Saudi Arabia; 6 Department of Biochemistry, King Abdulaziz University, Jeddah, Saudi Arabia; RMIT University, Australia

## Abstract

Over the past couple of decades there have been major advances in the field of nanoscience and nanotechnology. Many applications have sprouted from these fields of research. It is essential, given the scale of the materials, to attain accurate, valid and reproducible measurements. Material properties have shown to be a function of their size and composition. Physiochemical properties of the nanomaterials can significantly alter material behavior compared to bulk counterparts. For example, metal oxide nanoparticles have found broad applications ranging from photo-catalysis to antibacterial agents. In our study, we synthesized CuO nanoparticles using well established sol-gel based methods with varying levels of Ni doping. However, upon analysis of measured infrared data, we discovered the presence of quasi-periodic (QP) processes. Such processes have previously been reported to be tightly associated with measurement memory effects. We were able to detect the desired QP process in these measurements from three highly accurate repetitive experiments performed on each Ni (1–7%) doped CuO sample. In other words, successive measurements performed in a rather short period of time *remember* each other at least inside a group of neighboring measurements.

## Introduction

Copper oxide is a technologically important material which is mostly found in two oxidation states, cupric oxide (CuO) and cuprous oxide (Cu_2_O). Both are p-type semiconductors and the preferred structure of CuO is monoclinic, while that of Cu_2_O is cubic. The energy band gap of CuO ranges between 1.2–1.9 eV [1] and Cu_2_O has an energy band gap between 1.8–2.5 eV [2],[3]. As a result of some of the fascinating properties exhibited by copper oxide, it finds its application in high T_c_ superconductors [4], lithium ion electrode [5], gas sensors [6], [7], solar cells [8]–[10], field emission emitters [11]–[13], catalysis [14],[15], antibacterial agents [16] etc. Because CuO is a semiconductor, having a low band gap, it has also been applied for photoconductive and photothermal applications [9]. Nanomaterials, which have attracted major interest in recent years, possess a large amount of surfaces and interfaces and exhibit enhanced properties in comparison to their bulk counterparts. The properties of these materials can be further fine-tuned by controlling the particle sizes in the nano range or by playing with the crystal morphologies of the final product. Moreover, the doping of the semiconductor nanoparticles with impurity metal ions is one of the most important methods to modify the characteristics of the material. The engineering of band gap and influencing physical, chemical, and electronic properties of the semiconductors are possible by the use of the right amount of dopants. Several authors have reported the improvement in a material's properties using various dopants. The band gap narrowing of Ni doped SnO_2_ nanoparticles was observed by Ahmed et al. [17]. Das et al. reported the tuning of emission properties of Mn doped Cu_2_O nanoparticles [18]. Various methods have been developed for the synthesis of both pure and doped nanocrystalline CuO such as sol–gel method [19], one-step solid state reaction method [20], sonochemical method [21], electrochemical method [22], thermal decomposition of precursors [23] etc.. Out of these methods, sol–gel method is widely used for the synthesis of nanomaterials because of the various advantages associated with this method such as low cost, low temperatures processing, short annealing times, as well as higher purity of produced materials.

Inorganic metal oxides have also gained interest for applications as antimicrobial agents [16]. Advantages of using inorganic oxides such as TiO2, ZnO, and CuO are their stability, robustness, and long shelf life when compared with their organic counterparts [24]. A comprehensive analysis of the physiochemical properties of nanoparticles is essential for their characterization and for evaluating their activity. The determination of these properties is necessary to predict the fate, accumulation, and transport of nanoparticles based on surface and physiochemical characteristics [25]. FT-IR is a unique technique that offers a broad range of information about chemical structure and bonding of materials [26]. Many nanomaterial measurements, amongst them FT-IR, are sprinkled with fluctuations of different kinds that could actually be playing an important role. Conventional mathematical statistics could therefore be modified and tuned to analyze and overcome these unexpected properties providing more accurate and reliable information from different nanomaterial measurements. Previous methods such as procedure of the optimal linear smoothing (POLS) and probability distribution function (PDF) found that random sequences could help in data fitting parameters of well-known beta-distributions without a trend. [27]–[30].

In paper [27] we discovered that quasi-periodic (QP) processes are tightly associated with previous measured processes, thus having a memory. In other words, successive measurements performed in a rather short period of time *remember* each other at least inside a group of neighboring measurements. The detection of the QP processes in real measurements can significantly alter the existing conception of measurements. Experimentalists were accustomed to think that a group of repeated measurements should be independent from each other and, as a consequence of this supposition, the increasing number of experiments *N* decreases the dispersion as 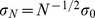 and thereby suppresses the influence of a “noise” (that are frequently associated with high-frequency fluctuations). The situation is completely changed if successive (repeatable) measurements have a memory. In accordance with a new conception considered in paper [27], the presence of memory between measurements mathematically can be expressed as
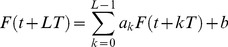
(1)


The solution of this functional equation has a form [27]

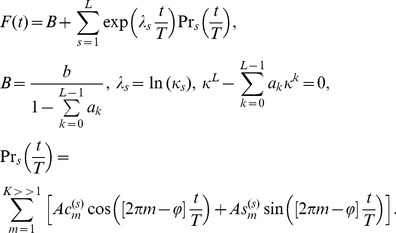
(2)


The fitting function *F*(*t*) was defined in [27] as the generalized Prony's decomposition and actually forms the generalized Prony's spectrum (GPS) because it can replace, approximately, at certain conditions the initial random function *y*(*t*) by a possible fitting function *F*(*t*) satisfying to equation (1). This function *F*(*t*) is determined by *K*(*L*+1)+2 number of parameters (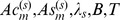) that form, in turn, the amplitude-frequency response (AFR) of the random function *y*(*t*) analyzed. The angle φ depends on the character of roots. In particular, for positive, negative and the complex-conjugated roots, the phase angle φ accepts the following values: 0, π, Im(λ)≠0, accordingly. For degenerated roots, this decomposition becomes more complicated. The view of decomposition function *F*(*t*) for this case is given in [27]. So, based on peculiarities of each experiment one can receive an *alternative* model, which admits the fitting of experimental functions to the function *F*(*t*) with the reduced set of fitting parameters.

Therefore, in this paper we want to demonstrate *new* possibilities for analysis of the IR spectra that were recorded for pure CuO with different concentrations of Ni (*c* = 1%,2%,3%,4%,5% and 7%). Each experiment was performed only three times. In spite of the minimal number of repetitions (equaled 3), the accuracy of each measurement was rather high that allowed detecting the desired QP process in these measurements.

## Materials and Methods

### Synthesis of pure and Ni doped CuO nanoparticles

Ni doped CuO nanoparticles with doping concentrations varying from 0% to7% were synthesized using previously established sol-gel based method [16]. For a typical synthesis procedure in distilled water dissolution of Cu(NO3)2.3H2O, Ni(NO3)2.6H2O and citric acid was carried out based on a molar ratio of 1-X:X:1 (where X represents the desired level of doping ranging 0.01–0.07). The solution was stirred at 80°C until gel formation (∼1 hr). Next, the gel was washed 2–3 times with ethanol using a microcentrifuge system at 19090 rcf to remove any organic impurities. Afterwards, the washed material in the form of dense precipitate was allowed to combust at 200°C for 10 minutes producing a light fluffy mass. After that, it was grinded for half an hour and further annealed at 350°C to obtain the highly crystalline Ni doped CuO nanoparticles. XDR, SEM and EDX analysis was carried out to confirm the presence of single phase materials and validated the level of doping present in each sample (supplementary material in Figures S1, S2, and S3).

### FT-IR Collection

FT-IR experiment was done at room temperature by an instrument from Thermo Scientific company (model name: Smart i TR). The instrument uses DTGS-KBr detector with beam splitter of KBr. Transmittance was measured with spectral data spacing of 0.964 cm^−1^ from 400 to 1000 cm^−1^. An atmospheric background was collected first followed by measuring each sample in triplicate before cleaning and measuring the next sample. The Ni doping level ranged from 0 to 7% within CuO nanoparticles.

## Results and Discussion

### Description of treatment procedure

In this section we want to demonstrate *how* to detect the QP process when the number of repetitions is small but accuracy of the reproduction of a current recording is rather high. In our specific case, the functional equation can have the following form
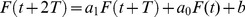
(3)


#### St1

**Figure 1:**
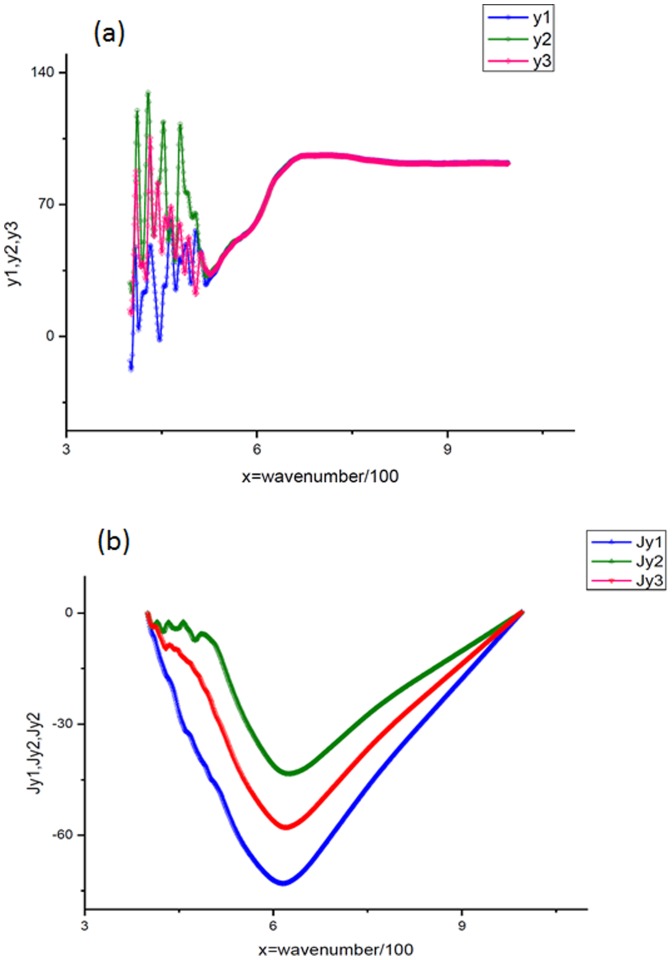
IR spectra of three pure CuO sample and their integrated curves. (a). The initial IR spectra corresponding to three successive measurements for pure CuO. The variable *x* here and below coincides with the normalized wavenumber/100 (*x* = λ/100). The intensity is given in some relative units. One can notice that random fluctuations located in the left-hand side destroy the picture of strong correlations between these three successive measurements. (b) The integrated curves that are obtained from the previous figure by means of expression (4). Now high-frequency fluctuations are suppressed and only the smoothed fluctuations in the left-hand side are noticeable.

The first step of the proposed algorithm began with the testing of equation (3). Let us consider these three independent measurements more attentively. The initial measurements depicted on Fig.1(a), and corresponding to pure CuO IR spectrum, look strongly-fluctuated. These “fluctuations” (located on the left-hand side) can hamper a possible link between measurements. A quite different picture is observed for their *integrated* curves, which are obtained from the initial ones by integration with respect to its mean value. The mathematical expression that helps to realize this procedure numerically can be expressed as
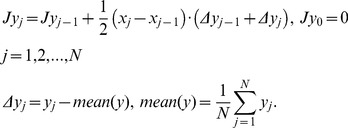
(4)


We want to stress here that *x*-variable coincides with the normalized value of wavenumber λ(cm^−1^)/100 and *y*-variable coincides with the relative intensity of the measured IR spectrum. One can pose two questions: (a) why the integration used as a smoothing procedure is chosen and (b) why is it necessary to integrate with respect to its mean value? Our answers are the following:

- (a). Any smoothing procedure (in spite of their varieties) and accuracy in extraction of the desired trend (see, for example, the most justified procedure designed for this purpose and defined as the optimal linear smoothing (POLS) procedure) [28]–[30]) creates some error. The integration does not disturb the initial fluctuations and extract only large-scale fluctuations, decreasing the value of the initial error without any uncontrollable procedure. That is why this direct integration procedure was chosen.
- (b). If we perform procedure (6) without subtraction of the value mean(*y*), then all large-scale fluctuations cannot be seen because of the essential contribution of a straight line ∼ *mean* (*y*)·*x* in the total integral. This contribution hides all these large-scale fluctuations.

The result of application of expression (6) is shown on Fig. 1(b). We obtain three similar integrated curves, and after that one can try to fit the third curve by a linear combination of two previous curves that belong to the first and second measurements, correspondingly. The result of this attempt is shown on Fig.1(c) As one can notice from this figure, the fitting curve describes the third integration curve with relatively high accuracy (the relative error is about 3.5%), and the supposition (3) is therefore correct. With the same success using the procedure outlined above, we checked the other 6 files corresponding to different concentrations of Ni (1%–5% and 7%). They also satisfy hypothesis (3) with different values of the constants (*a*_0,1_, *b*) figuring in (3). The calculation of the desired roots from equation
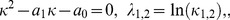
(5)shows that one root κ_1_ is always positive and another one κ_2_ is always negative for all 7 data files studied. In accordance with the general solution (2), these roots dictate the following structure of the solution *F*(*x*) that can be used as a fitting function for the integrated curves calculated 
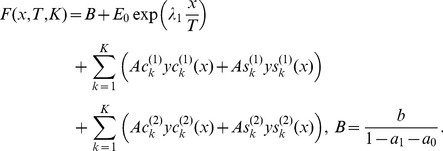
(6)


Here the functions 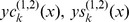
depend on the character of the roots λ_1,2_ = ln(κ_1,2_) (κ_1_>0, κ_2_<0). The calculated values of roots for all 7 files (pure CuO, (CuO+1%Ni)-(CuO+5%Ni) and CuO+7%Ni) are collected in Table 1.

**Table 1:** The values of the roots of equation (5) and the relative errors.

The values of the roots and the value of the fitting error	CuO	1%Ni	2%Ni	3%Ni	4%Ni	5%Ni	7%Ni
κ_2_	−0,40489	−0,67939	−0,05238	−0,9026	−0,0268	−0,35019	−0,87255
κ_1_	1,04759	1,03166	0,89394	0,97093	0,88119	1,03835	0,99516
RelErr(%)	3,50658	7,9868	9,88522	2,28185	2,81843	2,81477	0,70968

The values of the roots κ_2,1_ of equation (5) combined with the value of the relative error that fits equation (3), where the third integrated curve is presented as a linear combination of two previous integrated curves.



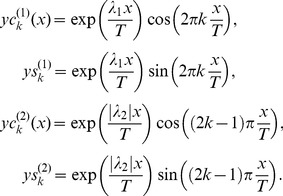
(7)


We should note that the independent variable *x* that enters into the last expressions does *not* coincide with real time (it coincides with the normalized wavenumber *x* = λ^−1^/100 as mentioned above). In this case, the value of a period *T* in expressions (6) and (7) cannot be fixed in experimental measurements and should be considered as *unknown* fitting parameter.

#### St2

**Figure 2:**
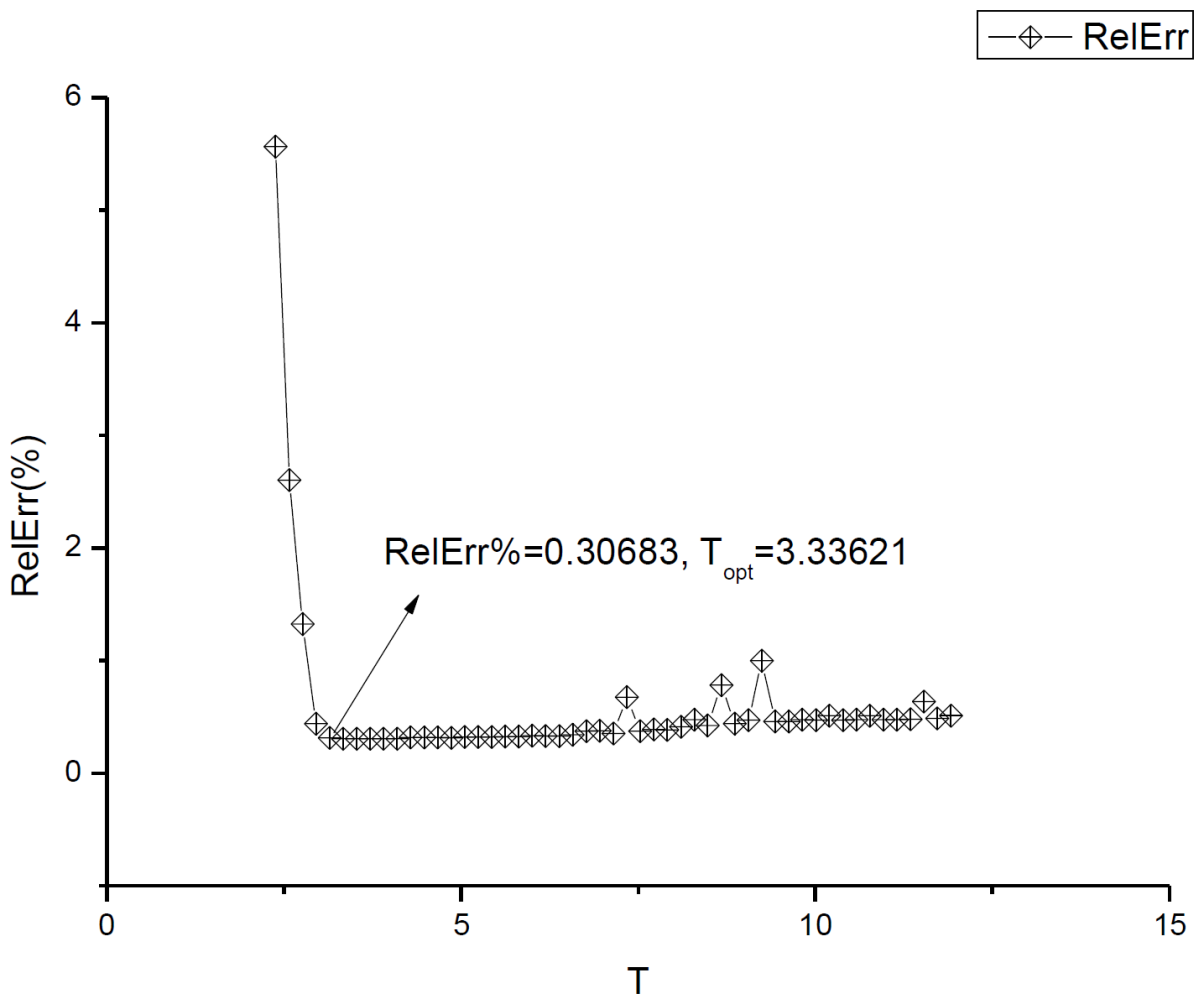
The procedure of the obtaining *T*_opt_. This plot explains the procedure of the finding the *T*_opt_ from expression [8]. It confirms also that the desired minimum is located in the interval [0.5*T*_max_, 2*T*_max_]. For all cases at the fixed value of *K* = 16 the value of the relative error does not exceed 1%.

For calculation of the unknown value *T*, it is necessary to find the limits of *T*. One can find the limits of this parameter from the following speculations. The minimal value of a “frequency” (in indirect sense) is defined from the following expression *f*_min_ = *h*⋅(length(*x*)), where *h* is the minimal value of discretization. So, the value of *T*_opt_ should be located in the vicinity of this value *T*_max_ = 1/*f*_min_ and can be less or it exceeds this value maximum at two times. So, we make a supposition that *T*_opt_ is located in the interval [0.5*T*_max_, 2*T*_max_]. The optimal value should correspond to the value of the minimal error and it is calculated from expression
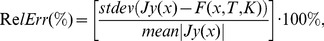
(8)where the integrated curve is calculated from expression (4) and the fitting function is taken from expression (6). The direct calculations confirm this supposition. Figure 2 demonstrates the dependence of the relative error (8) with respect to the value of *T* ∈ [0.5*T*_max_, 2*T*_max_] at the fixed value of *K*. The limiting value of *K* in decomposition (6) is fixed for all treated files (equaled 7) (*K* = 16) and chosen from the condition 0.1%<*RelErr*(%)<1% in order to provide a very accurate fit for the integrated curve corresponding to the third measurement.

#### St3

**Figure 3:**
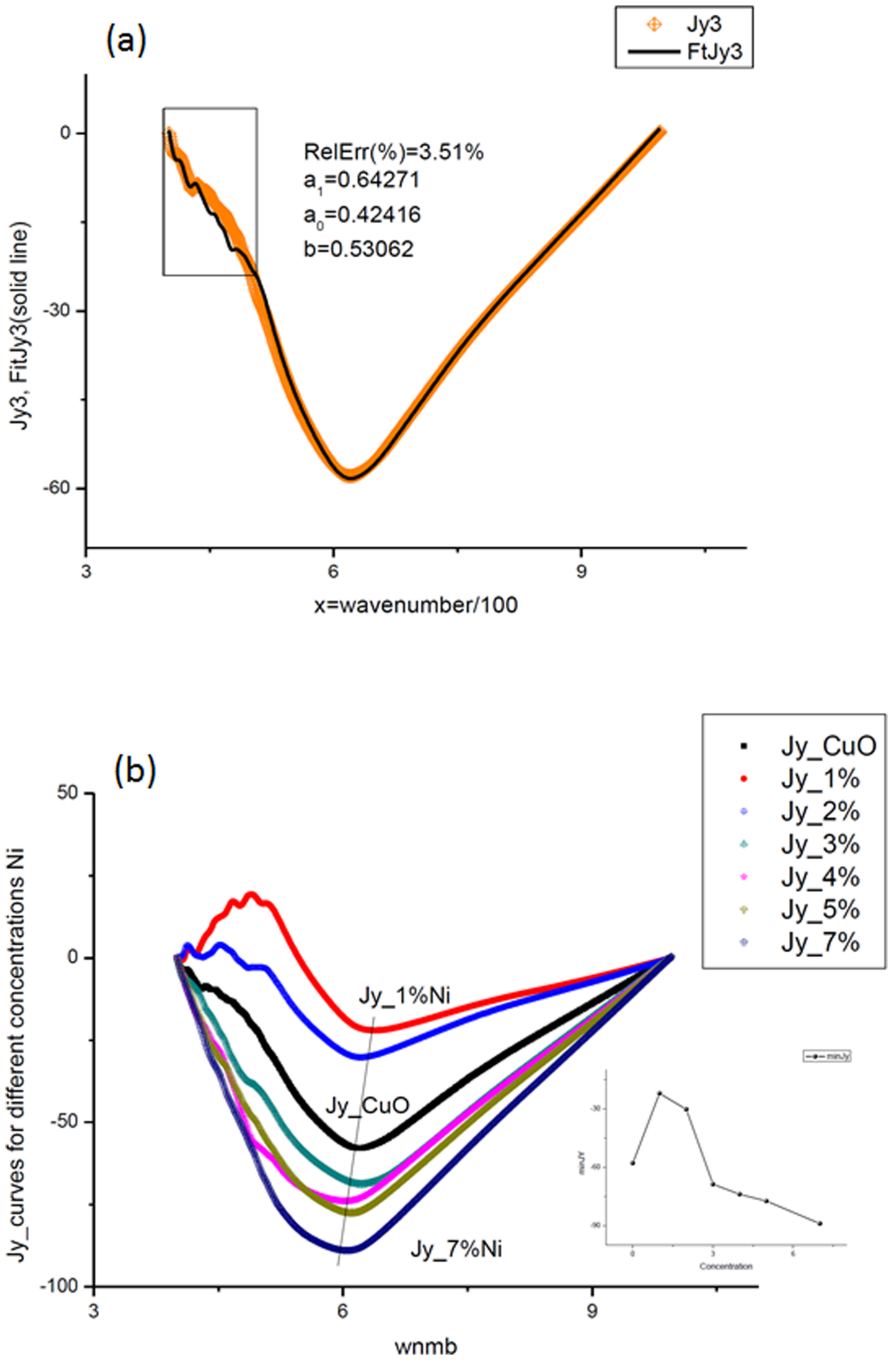
Hypothesis and integrated curve test results. (a). The results of the test of the hypothesis (3) is depicted on this figure. Linear combination of two previous measurements gives the acceptable fit (RelErr = 3.51%) of the integrated curve corresponding to the third measurement. The fitting constants from expression (3) are shown in the center of the figure. (b). This figure shows that integrated curves demonstrate the monotone behavior with respect to increasing of the doped Ni (1%→7%). More accurate behavior of this minimal point with respect to concentration is given in the small figure located on the right-hand side. We deliberately do not show the fitting curves to the function (6), which practically coincide with these curves. The value of the fitting error is located in the interval 0.1%<*RelErr*<1%.

**Figure 4:**
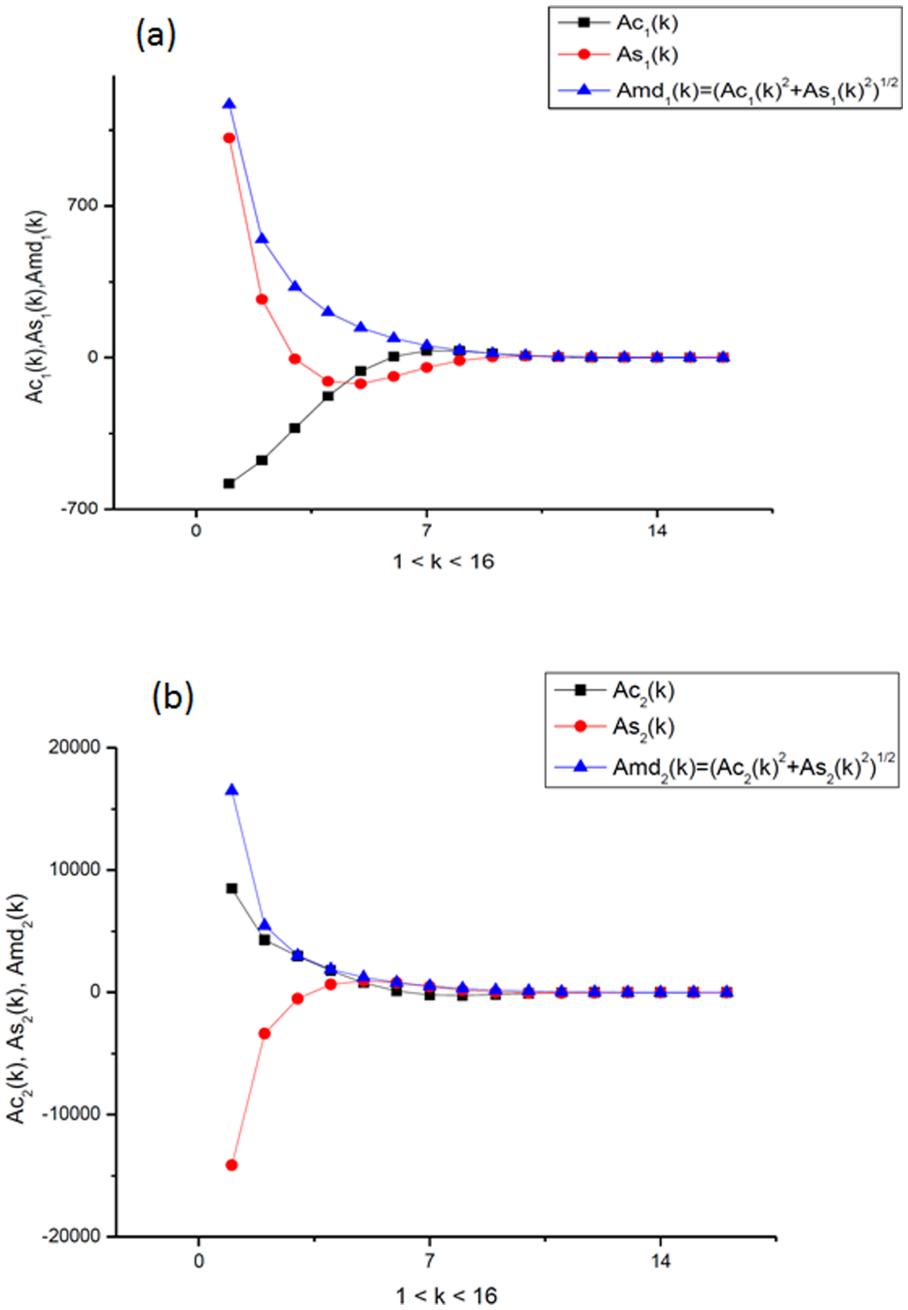
Amplitude-frequency responses. (a). The amplitude-frequency response that corresponds to the positive κ_1_>0 and describes the integrated curve corresponding to the third measurement of the IR spectrum of CuO. (b). The amplitude-frequency response that corresponds to the negative κ_2_<0 and describes the integrated curve corresponding to the third measurement of the IR spectrum of CuO.

This step can be considered as the final stage. After calculation of the nonlinear fitting parameter *T*_opt_ other linear fitting parameters (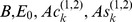) that enter to decomposition (6) are found by the linear-least square method (LLSM). The fit of the third integrated curve (corresponding to pure CuO) is shown in Fig. 3(a). Figure 3(b) shows the monotone decreasing of the minimal value of the integrated curves with increasing of concentration of Ni (1%→7%). This property can be used for calibration purposes. We deliberately do not show the perfect fit (that practically coincides with these curves) avoiding excess information. The value of the fitting error is collected in the last column of Table 2. The amplitude-frequency responses for the integrated curve CuO are shown in Figs 4(a, b). In the same manner we treated the remaining 6 files corresponding to different concentrations of Ni. The necessary parameters are collected in Table 2. Analysis of these preliminary results exhibits the following peculiarities that can be used for further analysis.

**Table 2:** The set of additional parameters that enter to the fitting function (6).

Concentration of Ni	*T*_opt_	λ_1_	*E*_0_	Range(Amd_1_(k))	Min(Jy_3_)	Relative Error(%)
		λ_2_	*B*	Range(Amd_2_(k))		
CuO	3,33621	0,0465	61260,1	885,233	−57,858	0,30483
		−0,90414	−68236,7	12617,8		
1%Ni	**3,71749**	0,03117	−1,55432E6	635643	−22,0667	0,79153
		−0,38656	1,3238E6	1,36998E6		
2%Ni	**3,71749**	−0,11212	84921,7	40592,8	−30,2731	0,35844
		−2,94925	−61361,2	8,54775E6		
3%Ni	**3,71749**	−0,0295	1,535E6	102623	−68,7515	0,15233
		−0,10248	−1,40664E6	118835		
4%Ni	**3,71749**	−0,12648	−365484	35550,5	−73,9134	0,15648
		−3,61936	258032	3,89219E7		
5%Ni	3,33621	0,03764	146140	1707,4	−77,3895	0,11271
		−1,04927	−159598	31878,8		
7%Ni	3,14557	−0,00485	−3053,68	11,4213	−88,9947	0,10808
		−0,13633	2974,13	48,476		

In column 1 we show the optimal values of the nonlinear fitting parameter *T_opt_*. The identical values are underlined and bolded.

In the 5-th column we give the ranges of the value 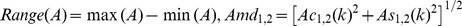
in order to stress large variations of this parameter in comparison with other parameters.

In the 6-th column we give the minimal value of the third integrated curve. It can be used for calibration purposes with respect to concentration of Ni.

1. The integrated curves clearly demonstrate the monotone decreasing of the minimal value with respect to the increasing of Ni concentrations. This peculiarity is obtained without application of any microscopic model and unnoticeable in original curves affected by strong fluctuations concentrated on the left hand-side.
2. Application of decomposition (6) allows reducing the information initially concentrated in 618 data points. After fitting we obtain only (4⋅*K*+3 = 67) fitting parameters that enter to the fitting function (6). This function reproduced the integrated curves with high accuracy.
3. The dependencies of the amplitudes 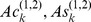 with respect to mode of *k* (*k* = 1,2,…,K) forms the generalized Prony's spectrum (GPS) and this spectrum can be read and compared with other data that are subjected to similar procedures. However, the third point cannot be realized because the detection of the QP processes is at the beginning.

We want to stress in conclusion the following important point. The presence of memory discovered in reproducible measurements can be considered a new direction of research in the near future. In any *ideal* experiment, all successive measurements do *not* remember each other. In other words, if we have a control variable *x* and *Pr*(*x*) represents a perfect response of the complex system studied then from the mathematical point of view the concept of *ideal* experiment should be expressed as
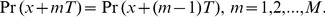
(9)


Here a letter *m = *1,2,…,*M* determines the number of successive measurements. As we can see from (9), the conventional Fourier transform receives a new and rather general interpretation and the decomposition coefficients of the periodic function 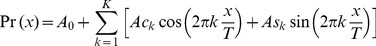
(10)
*A*_0_, *Ac_k_*, *As_k_* (*k* = 1,2,…,*K*) can be used for a quantitative expression of *ideal* measurements without memory. In reality we do *not* observe this situation. The strongly-correlated measurements (the integrated data in our case) demonstrate a memory, and instead of perfectly reproducible measurements without memory, we observe the opposite situation. Part of measurements remember each other and mathematical expression of this observation can be written as
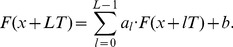
(11)


In our case we confirmed the partial case (3) of this general expression. Here the parameter *L* defines a length of a memory that can exist between neighboring measurements and the *memory coefficients a_l_* and *b* determine simultaneously the influence of the equipment and the object studied. This new and unexpected discovery that is contained in real measurements could help us to differentiate a device from an object. In other words, it will help to separate the Prony's decomposition (11) (aggravated by the uncontrollable factors from the equipment used) and be closer to the perfect case (9), where the *ideal* reproduction of data from the object studied could be solely present. These fine peculiarities discovered in real data will be important in experiments associated with nanotechnologies, where many “small” factors should be taken into account in order to provide stable and accurate measurements.

## Supporting Information

Figure S1 **FESEM image and EDX spectra (inset) of 4% Ni doped CuO nanoparticles confirming size and doping.**
(TIF)

Figure S2 **FESEM image and EDX spectra (inset) of 4% Ni doped CuO nanoparticles confirming size and doping.**
(TIF)

Figure S3 **XRD spectra of Ni doped (2% and 4%) CuO nanoparticles showing single phase spectra and lattices.**
(TIF)
