# Supplementary figures and images for: The First Observation of Memory Effects in the InfraRed (FT-IR) Measurements: Do Successive Measurements Remember Each Other?
Source: PLoS One. 2014 Apr 10;9(4):e94305. doi: 10.1371/journal.pone.0094305 (PMC3983122; doi:10.1371/journal.pone.0094305)

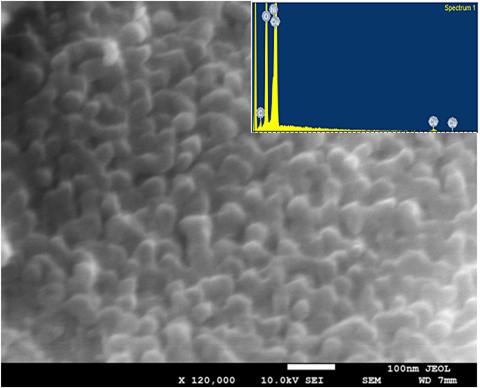

Supplement: Figure S1 — FESEM image and EDX spectra (inset) of 4% Ni doped CuO nanoparticles confirming size and doping. (TIF) [file pone.0094305.s001.tif]

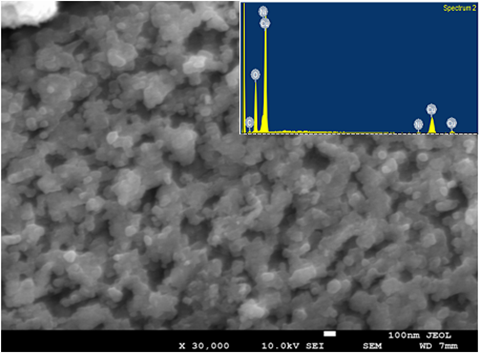

Supplement: Figure S2 — FESEM image and EDX spectra (inset) of 4% Ni doped CuO nanoparticles confirming size and doping. (TIF) [file pone.0094305.s002.tif]

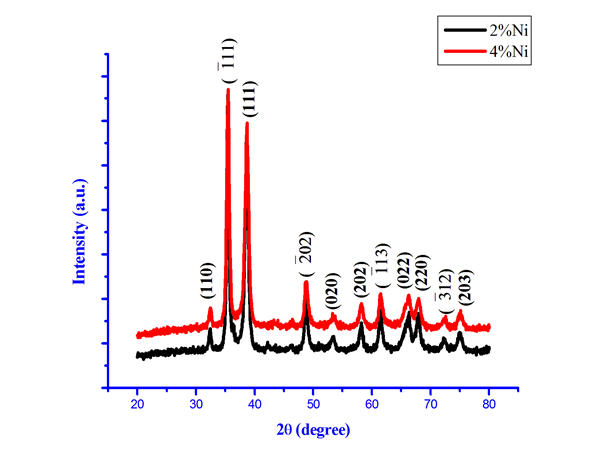

Supplement: Figure S3 — XRD spectra of Ni doped (2% and 4%) CuO nanoparticles showing single phase spectra and lattices. (TIF) [file pone.0094305.s003.tif]
